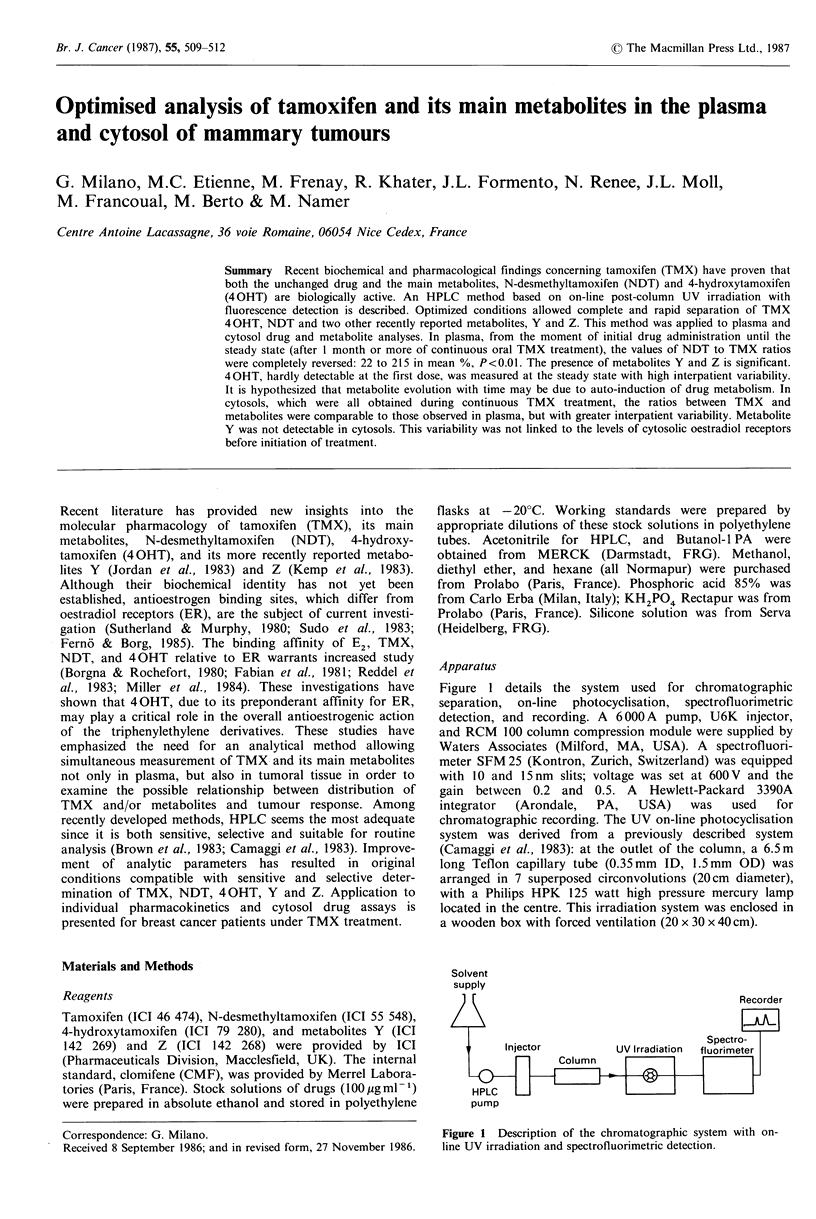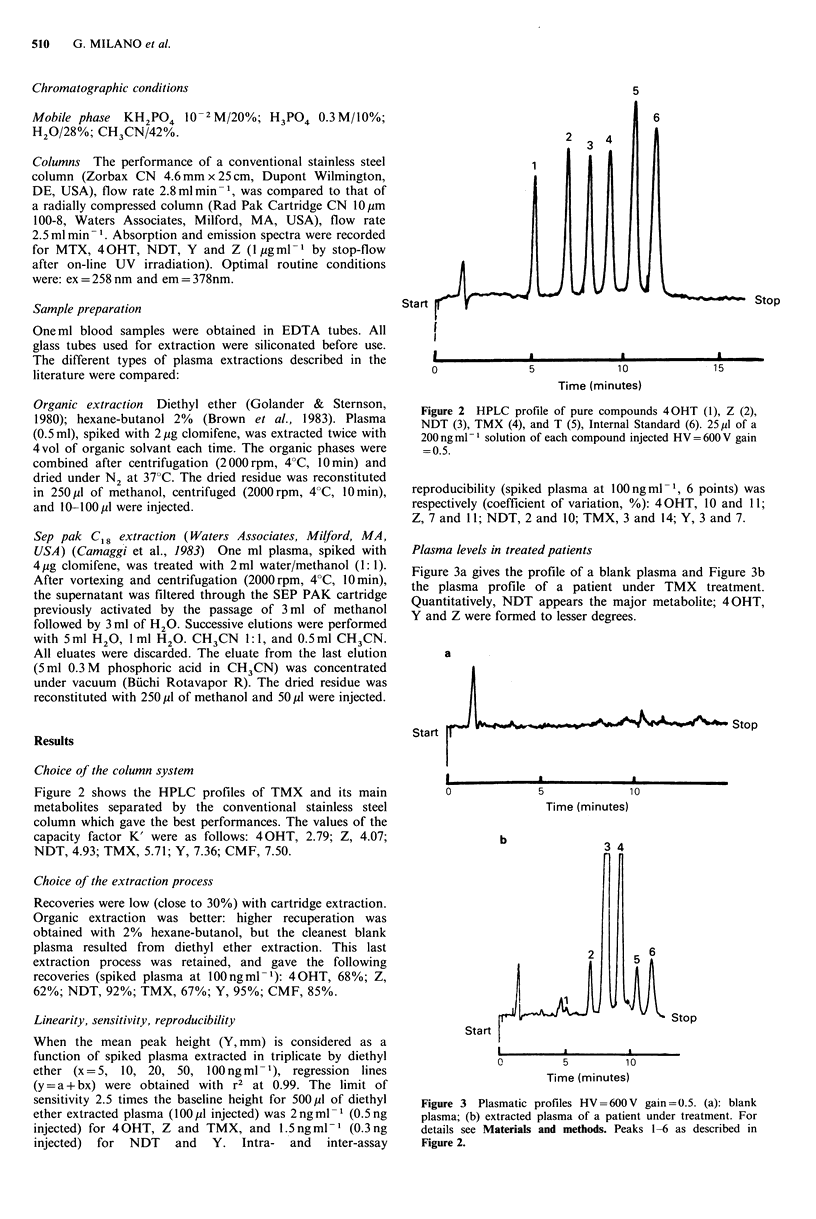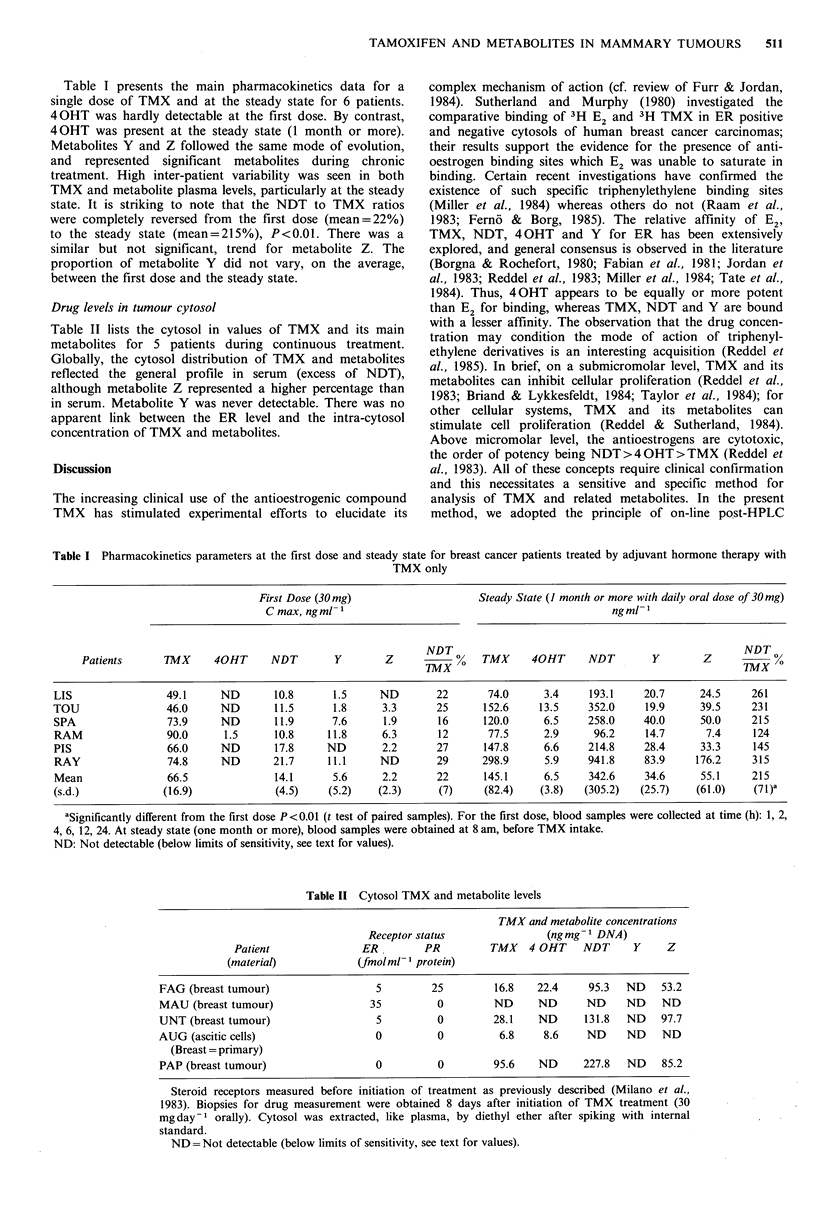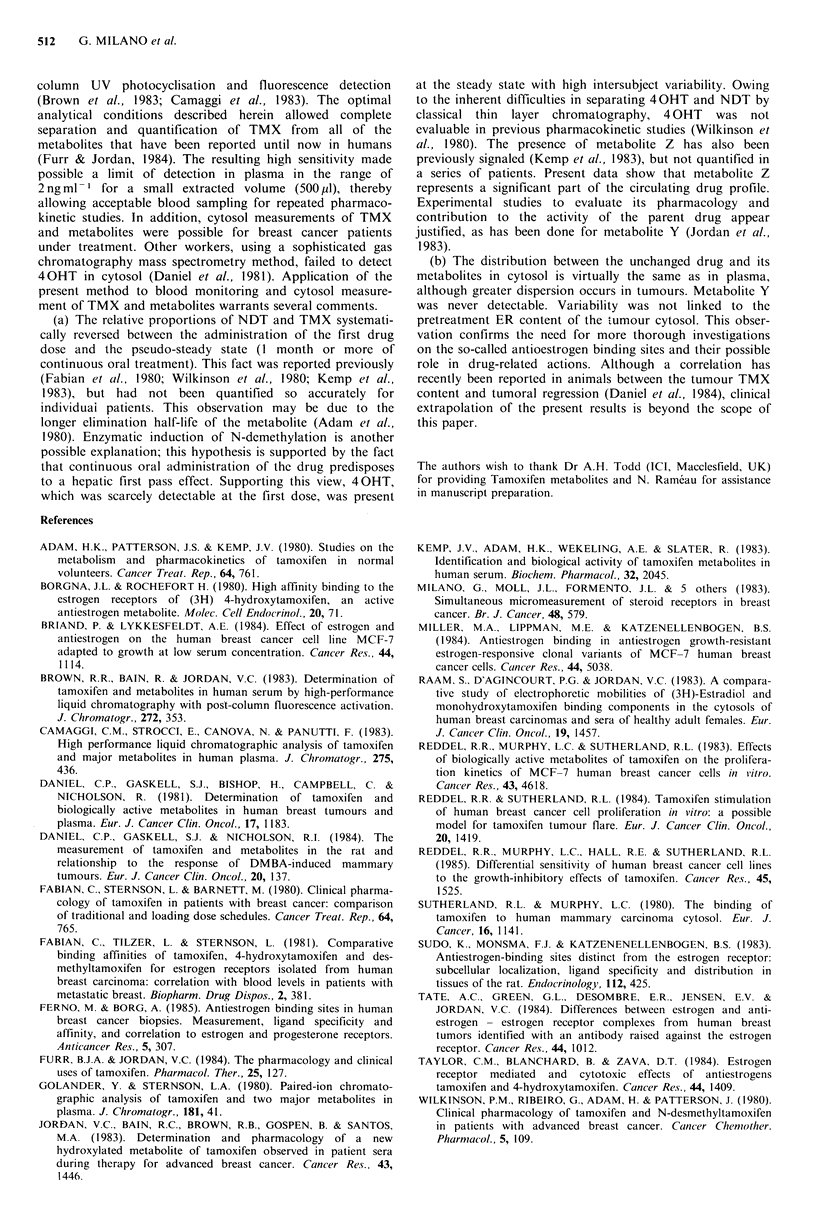# Optimised analysis of tamoxifen and its main metabolites in the plasma and cytosol of mammary tumours.

**DOI:** 10.1038/bjc.1987.103

**Published:** 1987-05

**Authors:** G. Milano, M. C. Etienne, M. Frenay, R. Khater, J. L. Formento, N. Renee, J. L. Moll, M. Francoual, M. Berto, M. Namer

## Abstract

Recent biochemical and pharmacological findings concerning tamoxifen (TMX) have proven that both the unchanged drug and the main metabolites, N-desmethyltamoxifen (NDT) and 4-hydroxytamoxifen (4OHT) are biologically active. An HPLC method based on on-line post-column UV irradiation with fluorescence detection is described. Optimized conditions allowed complete and rapid separation of TMX 4OHT, NDT and two other recently reported metabolites, Y and Z. This method was applied to plasma and cytosol drug and metabolite analyses. In plasma, from the moment of initial drug administration until the steady state (after 1 month or more of continuous oral TMX treatment), the values of NDT to TMX ratios were completely reversed: 22 to 215 in mean %, P less than 0.01. The presence of metabolites Y and Z is significant. 4OHT, hardly detectable at the first dose, was measured at the steady state with high interpatient variability. It is hypothesized that metabolite evolution with time may be due to auto-induction of drug metabolism. In cytosols, which were all obtained during continuous TMX treatment, the ratios between TMX and metabolites were comparable to those observed in plasma, but with greater interpatient variability. Metabolite Y was not detectable in cytosols. This variability was not linked to the levels of cytosolic oestradiol receptors before initiation of treatment.


*Br. J. Cancer* (1987), **55**, 509–512 © The Macmillan Press Ltd., 1987

### Optimised analysis of tamoxifen and its main metabolites in the plasma and cytosol of mammary tumours

G. Milano, M.C. Etienne, M. Frenay, R. Khater, J.L. Formento, N. Renee, J.L. Moll, M. Francoual, M. Berto & M. Namer

*Centre Antoine Lacassagne, 36 voie Romaine, 06054 Nice Cedex, France*

**Summary** Recent biochemical and pharmacological findings concerning tamoxifen (TMX) have proven that both the unchanged drug and the main metabolites, N-desmethyltamoxifen (NDT) and 4-hydroxytamoxifen (4OHT) are biologically active. An HPLC method based on on-line post-column UV irradiation with fluorescence detection is described. Optimized conditions allowed complete and rapid separation of TMX 4OHT, NDT and two other recently reported metabolites, Y and Z. This method was applied to plasma and cytosol drug and metabolite analyses. In plasma, from the moment of initial drug administration until the steady state (after 1 month or more of continuous oral TMX treatment), the values of NDT to TMX ratios were completely reversed: 22 to 215 in mean %, $P<0.01$. The presence of metabolites Y and Z is significant. 4OHT, hardly detectable at the first dose, was measured at the steady state with high interpatient variability. It is hypothesized that metabolite evolution with time may be due to auto-induction of drug metabolism. In cytosols, which were all obtained during continuous TMX treatment, the ratios between TMX and metabolites were comparable to those observed in plasma, but with greater interpatient variability. Metabolite Y was not detectable in cytosols. This variability was not linked to the levels of cytosolic oestradiol receptors before initiation of treatment.

Recent literature has provided new insights into the molecular pharmacology of tamoxifen (TMX), its main metabolites, N-desmethyltamoxifen (NDT), 4-hydroxytamoxifen (4OHT), and its more recently reported metabolites Y (Jordan *et al.*, 1983) and Z (Kemp *et al.*, 1983). Although their biochemical identity has not yet been established, antioestrogen binding sites, which differ from oestradiol receptors (ER), are the subject of current investigation (Sutherland & Murphy, 1980; Sudo *et al.*, 1983; Fernö & Borg, 1985). The binding affinity of $E_2$, TMX, NDT, and 4OHT relative to ER warrants increased study (Borgna & Rochefort, 1980; Fabian *et al.*, 1981; Reddel *et al.*, 1983; Miller *et al.*, 1984). These investigations have shown that 4OHT, due to its preponderant affinity for ER, may play a critical role in the overall antioestrogenic action of the triphenylethylene derivatives. These studies have emphasized the need for an analytical method allowing simultaneous measurement of TMX and its main metabolites not only in plasma, but also in tumoral tissue in order to examine the possible relationship between distribution of TMX and/or metabolites and tumour response. Among recently developed methods, HPLC seems the most adequate since it is both sensitive, selective and suitable for routine analysis (Brown *et al.*, 1983; Camaggi *et al.*, 1983). Improvement of analytic parameters has resulted in original conditions compatible with sensitive and selective determination of TMX, NDT, 4OHT, Y and Z. Application to individual pharmacokinetics and cytosol drug assays is presented for breast cancer patients under TMX treatment.

### Materials and Methods

### *Reagents*

Tamoxifen (ICI 46 474), N-desmethyltamoxifen (ICI 55 548), 4-hydroxytamoxifen (ICI 79 280), and metabolites Y (ICI 142 269) and Z (ICI 142 268) were provided by ICI (Pharmaceuticals Division, Macclesfield, UK). The internal standard, clomifene (CMF), was provided by Merrel Laboratories (Paris, France). Stock solutions of drugs ($100\,\mu g\,ml^{-1}$) were prepared in absolute ethanol and stored in polyethylene flasks at −20°C. Working standards were prepared by appropriate dilutions of these stock solutions in polyethylene tubes. Acetonitrile for HPLC, and Butanol-1 PA were obtained from MERCK (Darmstadt, FRG). Methanol, diethyl ether, and hexane (all Normapur) were purchased from Prolabo (Paris, France). Phosphoric acid 85% was from Carlo Erba (Milan, Italy); $KH_2PO_4$ Rectapur was from Prolabo (Paris, France). Silicone solution was from Serva (Heidelberg, FRG).

Correspondence: G. Milano.
Received 8 September 1986; and in revised form, 27 November 1986.

### *Apparatus*

Figure 1 details the system used for chromatographic separation, on-line photocyclisation, spectrofluorimetric detection, and recording. A 6000A pump, U6K injector, and RCM 100 column compression module were supplied by Waters Associates (Milford, MA, USA). A spectrofluorimeter SFM25 (Kontron, Zurich, Switzerland) was equipped with 10 and 15 nm slits; voltage was set at 600 V and the gain between 0.2 and 0.5. A Hewlett-Packard 3390A integrator (Arondale, PA, USA) was used for chromatographic recording. The UV on-line photocyclisation system was derived from a previously described system (Camaggi *et al.*, 1983): at the outlet of the column, a 6.5 m long Teflon capillary tube (0.35 mm ID, 1.5 mm OD) was arranged in 7 superposed circonvolutions (20 cm diameter), with a Philips HPK 125 watt high pressure mercury lamp located in the centre. This irradiation system was enclosed in a wooden box with forced ventilation (20 x 30 x 40 cm).

**Figure 1** Description of the chromatographic system with on-line UV irradiation and spectrofluorimetric detection.

*Chromatographic conditions*

*Mobile phase* $KH_2PO_4$ $10^{-2}$ M/20%; $H_3PO_4$ 0.3 M/10%; $H_2O$/28%; $CH_3CN$/42%.

*Columns* The performance of a conventional stainless steel column (Zorbax CN 4.6 mm x 25 cm, Dupont Wilmington, DE, USA), flow rate 2.8 ml min$^{-1}$, was compared to that of a radially compressed column (Rad Pak Cartridge CN 10 $\mu$m 100-8, Waters Associates, Milford, MA, USA), flow rate 2.5 ml min$^{-1}$. Absorption and emission spectra were recorded for MTX, 4OHT, NDT, Y and Z (1 $\mu$g ml$^{-1}$ by stop-flow after on-line UV irradiation). Optimal routine conditions were: ex = 258 nm and em = 378nm.

*Sample preparation*

One ml blood samples were obtained in EDTA tubes. All glass tubes used for extraction were siliconated before use. The different types of plasma extractions described in the literature were compared:

*Organic extraction* Diethyl ether (Golander & Sternson, 1980); hexane-butanol 2% (Brown *et al.*, 1983). Plasma (0.5 ml), spiked with 2 $\mu$g clomifene, was extracted twice with 4 vol of organic solvant each time. The organic phases were combined after centrifugation (2 000 rpm, 4°C, 10 min) and dried under $N_2$ at 37°C. The dried residue was reconstituted in 250 $\mu$l of methanol, centrifuged (2000 rpm, 4°C, 10 min), and 10–100 $\mu$l were injected.

*Sep pak $C_{18}$ extraction (Waters Associates, Milford, MA, USA) (Camaggi* et al., *1983)* One ml plasma, spiked with 4 $\mu$g clomifene, was treated with 2 ml water/methanol (1:1). After vortexing and centrifugation (2000 rpm, 4°C, 10 min), the supernatant was filtered through the SEP PAK cartridge previously activated by the passage of 3 ml of methanol followed by 3 ml of $H_2O$. Successive elutions were performed with 5 ml $H_2O$, 1 ml $H_2O$. $CH_3CN$ 1:1, and 0.5 ml $CH_3CN$. All eluates were discarded. The eluate from the last elution (5 ml 0.3 M phosphoric acid in $CH_3CN$) was concentrated under vacuum (Büchi Rotavapor R). The dried residue was reconstituted with 250 $\mu$l of methanol and 50 $\mu$l were injected.

### Results

*Choice of the column system*

Figure 2 shows the HPLC profiles of TMX and its main metabolites separated by the conventional stainless steel column which gave the best performances. The values of the capacity factor K′ were as follows: 4OHT, 2.79; Z, 4.07; NDT, 4.93; TMX, 5.71; Y, 7.36; CMF, 7.50.

*Choice of the extraction process*

Recoveries were low (close to 30%) with cartridge extraction. Organic extraction was better: higher recuperation was obtained with 2% hexane-butanol, but the cleanest blank plasma resulted from diethyl ether extraction. This last extraction process was retained, and gave the following recoveries (spiked plasma at 100 ng ml$^{-1}$): 4OHT, 68%; Z, 62%; NDT, 92%; TMX, 67%; Y, 95%; CMF, 85%.

*Linearity, sensitivity, reproducibility*

When the mean peak height (Y, mm) is considered as a function of spiked plasma extracted in triplicate by diethyl ether (x = 5, 10, 20, 50, 100 ng ml$^{-1}$), regression lines (y = a + bx) were obtained with $r^2$ at 0.99. The limit of sensitivity 2.5 times the baseline height for 500 $\mu$l of diethyl ether extracted plasma (100 $\mu$l injected) was 2 ng ml$^{-1}$ (0.5 ng injected) for 4OHT, Z and TMX, and 1.5 ng ml$^{-1}$ (0.3 ng injected) for NDT and Y. Intra- and inter-assay reproducibility (spiked plasma at 100 ng ml$^{-1}$, 6 points) was respectively (coefficient of variation, %): 4OHT, 10 and 11; Z, 7 and 11; NDT, 2 and 10; TMX, 3 and 14; Y, 3 and 7.

**Figure 2** HPLC profile of pure compounds 4OHT (1), Z (2), NDT (3), TMX (4), and T (5), Internal Standard (6). 25 $\mu$l of a 200 ng ml$^{-1}$ solution of each compound injected HV = 600 V gain = 0.5.

*Plasma levels in treated patients*

Figure 3a gives the profile of a blank plasma and Figure 3b the plasma profile of a patient under TMX treatment. Quantitatively, NDT appears the major metabolite; 4OHT, Y and Z were formed to lesser degrees.

**Figure 3** Plasmatic profiles HV = 600 V gain = 0.5. (a): blank plasma; (b) extracted plasma of a patient under treatment. For details see **Materials and methods.** Peaks 1–6 as described in **Figure 2.**

Table I presents the main pharmacokinetics data for a single dose of TMX and at the steady state for 6 patients. 4OHT was hardly detectable at the first dose. By contrast, 4OHT was present at the steady state (1 month or more). Metabolites Y and Z followed the same mode of evolution, and represented significant metabolites during chronic treatment. High inter-patient variability was seen in both TMX and metabolite plasma levels, particularly at the steady state. It is striking to note that the NDT to TMX ratios were completely reversed from the first dose (mean = 22%) to the steady state (mean = 215%), $P<0.01$. There was a similar but not significant, trend for metabolite Z. The proportion of metabolite Y did not vary, on the average, between the first dose and the steady state.

*Drug levels in tumour cytosol*

Table II lists the cytosol in values of TMX and its main metabolites for 5 patients during continuous treatment. Globally, the cytosol distribution of TMX and metabolites reflected the general profile in serum (excess of NDT), although metabolite Z represented a higher percentage than in serum. Metabolite Y was never detectable. There was no apparent link between the ER level and the intra-cytosol concentration of TMX and metabolites.

### Discussion

The increasing clinical use of the antioestrogenic compound TMX has stimulated experimental efforts to elucidate its complex mechanism of action (cf. review of Furr & Jordan, 1984). Sutherland and Murphy (1980) investigated the comparative binding of $^3H$ $E_2$ and $^3H$ TMX in ER positive and negative cytosols of human breast cancer carcinomas; their results support the evidence for the presence of anti-oestrogen binding sites which $E_2$ was unable to saturate in binding. Certain recent investigations have confirmed the existence of such specific triphenylethylene binding sites (Miller *et al.*, 1984) whereas others do not (Raam *et al.*, 1983; Fernö & Borg, 1985). The relative affinity of $E_2$, TMX, NDT, 4OHT and Y for ER has been extensively explored, and general consensus is observed in the literature (Borgna & Rochefort, 1980; Fabian *et al.*, 1981; Jordan *et al.*, 1983; Reddel *et al.*, 1983; Miller *et al.*, 1984; Tate *et al.*, 1984). Thus, 4OHT appears to be equally or more potent than $E_2$ for binding, whereas TMX, NDT and Y are bound with a lesser affinity. The observation that the drug concentration may condition the mode of action of triphenylethylene derivatives is an interesting acquisition (Reddel *et al.*, 1985). In brief, on a submicromolar level, TMX and its metabolites can inhibit cellular proliferation (Reddel *et al.*, 1983; Briand & Lykkesfeldt, 1984; Taylor *et al.*, 1984); for other cellular systems, TMX and its metabolites can stimulate cell proliferation (Reddel & Sutherland, 1984). Above micromolar level, the antioestrogens are cytotoxic, the order of potency being NDT > 4OHT > TMX (Reddel *et al.*, 1983). All of these concepts require clinical confirmation and this necessitates a sensitive and specific method for analysis of TMX and related metabolites. In the present method, we adopted the principle of on-line post-HPLC

**Table I** Pharmacokinetics parameters at the first dose and steady state for breast cancer patients treated by adjuvant hormone therapy with TMX only

| | *First Dose (30 mg) C max, ng ml$^{-1}$* | | | | | | *Steady State (1 month or more with daily oral dose of 30 mg) ng ml$^{-1}$* | | | | | |
|---|---|---|---|---|---|---|---|---|---|---|---|---|
| *Patients* | *TMX* | *4OHT* | *NDT* | *Y* | *Z* | $\frac{NDT}{TMX}$ % | *TMX* | *4OHT* | *NDT* | *Y* | *Z* | $\frac{NDT}{TMX}$ % |
| LIS | 49.1 | ND | 10.8 | 1.5 | ND | 22 | 74.0 | 3.4 | 193.1 | 20.7 | 24.5 | 261 |
| TOU | 46.0 | ND | 11.5 | 1.8 | 3.3 | 25 | 152.6 | 13.5 | 352.0 | 19.9 | 39.5 | 231 |
| SPA | 73.9 | ND | 11.9 | 7.6 | 1.9 | 16 | 120.0 | 6.5 | 258.0 | 40.0 | 50.0 | 215 |
| RAM | 90.0 | 1.5 | 10.8 | 11.8 | 6.3 | 12 | 77.5 | 2.9 | 96.2 | 14.7 | 7.4 | 124 |
| PIS | 66.0 | ND | 17.8 | ND | 2.2 | 27 | 147.8 | 6.6 | 214.8 | 28.4 | 33.3 | 145 |
| RAY | 74.8 | ND | 21.7 | 11.1 | ND | 29 | 298.9 | 5.9 | 941.8 | 83.9 | 176.2 | 315 |
| Mean | 66.5 | | 14.1 | 5.6 | 2.2 | 22 | 145.1 | 6.5 | 342.6 | 34.6 | 55.1 | 215 |
| (s.d.) | (16.9) | | (4.5) | (5.2) | (2.3) | (7) | (82.4) | (3.8) | (305.2) | (25.7) | (61.0) | (71)[a] |

[a]Significantly different from the first dose $P<0.01$ (*t* test of paired samples). For the first dose, blood samples were collected at time (h): 1, 2, 4, 6, 12, 24. At steady state (one month or more), blood samples were obtained at 8 am, before TMX intake.
ND: Not detectable (below limits of sensitivity, see text for values).

**Table II** Cytosol TMX and metabolite levels

| *Patient (material)* | *Receptor status ER (fmol ml$^{-1}$ protein)* | *PR* | *TMX and metabolite concentrations (ng mg$^{-1}$ DNA) TMX* | *4 OHT* | *NDT* | *Y* | *Z* |
|---|---|---|---|---|---|---|---|
| FAG (breast tumour) | 5 | 25 | 16.8 | 22.4 | 95.3 | ND | 53.2 |
| MAU (breast tumour) | 35 | 0 | ND | ND | ND | ND | ND |
| UNT (breast tumour) | 5 | 0 | 28.1 | ND | 131.8 | ND | 97.7 |
| AUG (ascitic cells) (Breast = primary) | 0 | 0 | 6.8 | 8.6 | ND | ND | ND |
| PAP (breast tumour) | 0 | 0 | 95.6 | ND | 227.8 | ND | 85.2 |

Steroid receptors measured before initiation of treatment as previously described (Milano *et al.*, 1983). Biopsies for drug measurement were obtained 8 days after initiation of TMX treatment (30 mg day$^{-1}$ orally). Cytosol was extracted, like plasma, by diethyl ether after spiking with internal standard.
ND = Not detectable (below limits of sensitivity, see text for values).

column UV photocyclisation and fluorescence detection (Brown *et al.*, 1983; Camaggi *et al.*, 1983). The optimal analytical conditions described herein allowed complete separation and quantification of TMX from all of the metabolites that have been reported until now in humans (Furr & Jordan, 1984). The resulting high sensitivity made possible a limit of detection in plasma in the range of $2\,ng\,ml^{-1}$ for a small extracted volume ($500\,\mu l$), thereby allowing acceptable blood sampling for repeated pharmacokinetic studies. In addition, cytosol measurements of TMX and metabolites were possible for breast cancer patients under treatment. Other workers, using a sophisticated gas chromatography mass spectrometry method, failed to detect 4OHT in cytosol (Daniel *et al.*, 1981). Application of the present method to blood monitoring and cytosol measurement of TMX and metabolites warrants several comments.

(a) The relative proportions of NDT and TMX systematically reversed between the administration of the first drug dose and the pseudo-steady state (1 month or more of continuous oral treatment). This fact was reported previously (Fabian *et al.*, 1980; Wilkinson *et al.*, 1980; Kemp *et al.*, 1983), but had not been quantified so accurately for individuai patients. This observation may be due to the longer elimination half-life of the metabolite (Adam *et al.*, 1980). Enzymatic induction of N-demethylation is another possible explanation; this hypothesis is supported by the fact that continuous oral administration of the drug predisposes to a hepatic first pass effect. Supporting this view, 4OHT, which was scarcely detectable at the first dose, was present at the steady state with high intersubject variability. Owing to the inherent difficulties in separating 4OHT and NDT by classical thin layer chromatography, 4OHT was not evaluable in previous pharmacokinetic studies (Wilkinson *et al.*, 1980). The presence of metabolite Z has also been previously signaled (Kemp *et al.*, 1983), but not quantified in a series of patients. Present data show that metabolite Z represents a significant part of the circulating drug profile. Experimental studies to evaluate its pharmacology and contribution to the activity of the parent drug appear justified, as has been done for metabolite Y (Jordan *et al.*, 1983).

(b) The distribution between the unchanged drug and its metabolites in cytosol is virtually the same as in plasma, although greater dispersion occurs in tumours. Metabolite Y was never detectable. Variability was not linked to the pretreatment ER content of the tumour cytosol. This observation confirms the need for more thorough investigations on the so-called antioestrogen binding sites and their possible role in drug-related actions. Although a correlation has recently been reported in animals between the tumour TMX content and tumoral regression (Daniel *et al.*, 1984), clinical extrapolation of the present results is beyond the scope of this paper.

The authors wish to thank Dr A.H. Todd (ICI, Macclesfield, UK) for providing Tamoxifen metabolites and N. Raméau for assistance in manuscript preparation.

### References

ADAM, H.K., PATTERSON, J.S. & KEMP, J.V. (1980). Studies on the metabolism and pharmacokinetics of tamoxifen in normal volunteers. *Cancer Treat. Rep.*, **64,** 761.

BORGNA, J.L. & ROCHEFORT H. (1980). High affinity binding to the estrogen receptors of (3H) 4-hydroxytamoxifen, an active antiestrogen metabolite. *Molec. Cell Endocrinol.*, **20,** 71.

BRIAND, P. & LYKKESFELDT, A.E. (1984). Effect of estrogen and antiestrogen on the human breast cancer cell line MCF-7 adapted to growth at low serum concentration. *Cancer Res.*, **44,** 1114.

BROWN, R.R., BAIN, R. & JORDAN, V.C. (1983). Determination of tamoxifen and metabolites in human serum by high-performance liquid chromatography with post-column fluorescence activation. *J. Chromatogr.*, **272,** 353.

CAMAGGI, C.M., STROCCI, E., CANOVA, N. & PANUTTI, F. (1983). High performance liquid chromatographic analysis of tamoxifen and major metabolites in human plasma. *J. Chromatogr.*, **275,** 436.

DANIEL, C.P., GASKELL, S.J., BISHOP, H., CAMPBELL, C. & NICHOLSON, R. (1981). Determination of tamoxifen and biologically active metabolites in human breast tumours and plasma. *Eur. J. Cancer Clin. Oncol.*, **17,** 1183.

DANIEL, C.P., GASKELL, S.J. & NICHOLSON, R.I. (1984). The measurement of tamoxifen and metabolites in the rat and relationship to the response of DMBA-induced mammary tumours. *Eur. J. Cancer Clin. Oncol.*, **20,** 137.

FABIAN, C., STERNSON, L. & BARNETT, M. (1980). Clinical pharmacology of tamoxifen in patients with breast cancer: comparison of traditional and loading dose schedules. *Cancer Treat. Rep.*, **64,** 765.

FABIAN, C., TILZER, L. & STERNSON, L. (1981). Comparative binding affinities of tamoxifen, 4-hydroxytamoxifen and desmethyltamoxifen for estrogen receptors isolated from human breast carcinoma: correlation with blood levels in patients with metastatic breast. *Biopharm. Drug Dispos.*, **2,** 381.

FERNO, M. & BORG, A. (1985). Antiestrogen binding sites in human breast cancer biopsies. Measurement, ligand specificity and affinity, and correlation to estrogen and progesterone receptors. *Anticancer Res.*, **5,** 307.

FURR, B.J.A. & JORDAN, V.C. (1984). The pharmacology and clinical uses of tamoxifen. *Pharmacol. Ther.*, **25,** 127.

GOLANDER, Y. & STERNSON, L.A. (1980). Paired-ion chromatographic analysis of tamoxifen and two major metabolites in plasma. *J. Chromatogr.*, **181,** 41.

JORDAN, V.C., BAIN, R.C., BROWN, R.B., GOSPEN, B. & SANTOS, M.A. (1983). Determination and pharmacology of a new hydroxylated metabolite of tamoxifen observed in patient sera during therapy for advanced breast cancer. *Cancer Res.*, **43,** 1446.

KEMP, J.V., ADAM, H.K., WEKELING, A.E. & SLATER, R. (1983). Identification and biological activity of tamoxifen metabolites in human serum. *Biochem. Pharmacol.*, **32,** 2045.

MILANO, G., MOLL, J.L., FORMENTO, J.L. & 5 others (1983). Simultaneous micromeasurement of steroid receptors in breast cancer. *Br. J. Cancer*, **48,** 579.

MILLER, M.A., LIPPMAN, M.E. & KATZENELLENBOGEN, B.S. (1984). Antiestrogen binding in antiestrogen growth-resistant estrogen-responsive clonal variants of MCF–7 human breast cancer cells. *Cancer Res.*, **44,** 5038.

RAAM, S., D'AGINCOURT, P.G. & JORDAN, V.C. (1983). A comparative study of electrophoretic mobilities of (3H)-Estradiol and monohydroxytamoxifen binding components in the cytosols of human breast carcinomas and sera of healthy adult females. *Eur. J. Cancer Clin. Oncol.*, **19,** 1457.

REDDEL, R.R., MURPHY, L.C. & SUTHERLAND, R.L. (1983). Effects of biologically active metabolites of tamoxifen on the proliferation kinetics of MCF–7 human breast cancer cells *in vitro*. *Cancer Res.*, **43,** 4618.

REDDEL, R.R. & SUTHERLAND, R.L. (1984). Tamoxifen stimulation of human breast cancer cell proliferation *in vitro*: a possible model for tamoxifen tumour flare. *Eur. J. Cancer Clin. Oncol.*, **20,** 1419.

REDDEL, R.R., MURPHY, L.C., HALL, R.E. & SUTHERLAND, R.L. (1985). Differential sensitivity of human breast cancer cell lines to the growth-inhibitory effects of tamoxifen. *Cancer Res.*, **45,** 1525.

SUTHERLAND, R.L. & MURPHY, L.C. (1980). The binding of tamoxifen to human mammary carcinoma cytosol. *Eur. J. Cancer*, **16,** 1141.

SUDO, K., MONSMA, F.J. & KATZENENELLENBOGEN, B.S. (1983). Antiestrogen-binding sites distinct from the estrogen receptor: subcellular localization, ligand specificity and distribution in tissues of the rat. *Endocrinology*, **112,** 425.

TATE, A.C., GREEN, G.L., DESOMBRE, E.R., JENSEN, E.V. & JORDAN, V.C. (1984). Differences between estrogen and anti-estrogen – estrogen receptor complexes from human breast tumors identified with an antibody raised against the estrogen receptor. *Cancer Res.*, **44,** 1012.

TAYLOR, C.M., BLANCHARD, B. & ZAVA, D.T. (1984). Estrogen receptor mediated and cytotoxic effects of antiestrogens tamoxifen and 4-hydroxytamoxifen. *Cancer Res.*, **44,** 1409.

WILKINSON, P.M., RIBEIRO, G., ADAM, H. & PATTERSON, J. (1980). Clinical pharmacology of tamoxifen and N-desmethyltamoxifen in patients with advanced breast cancer. *Cancer Chemother. Pharmacol.*, **5,** 109.